# The prevalence of hypothyroxinemia in premature newborns

**DOI:** 10.3389/fendo.2022.940152

**Published:** 2022-08-10

**Authors:** Renata Stawerska, Marzena Nowak-Bednarek, Tomasz Talar, Marzena Kolasa-Kicińska, Anna Łupińska, Maciej Hilczer, Ewa Gulczyńska, Andrzej Lewiński

**Affiliations:** ^1^ Department of Endocrinology and Metabolic Diseases, Polish Mother’s Memorial - Hospital Research Institute, Lodz, Poland; ^2^ Department of Paediatric Endocrinology, Medical University of Lodz, Lodz, Poland; ^3^ Department of Neonatology, Intensive Therapy and Neonatal Pathology, Polish Mother’s Memorial Hospital – Research Institute, Lodz, Poland; ^4^ Department of Endocrinology and Metabolic Diseases, Medical University of Lodz, Lodz, Poland

**Keywords:** congenital hypothyroidism, hypothyroxinemia, neonatal screening, preterm newborns, small for gestational age, thyroid hormones, thyroid stimulating hormone

## Abstract

Congenital hypothyroidism diagnosed by TSH assessment in bloodspot screening may be overlooked in preterm newborns due to immaturity of the hypothalamus-pituitary-thyroid axis in them. The purpose of the study was to determine the prevalence and causes of hypothyroxinemia in preterm newborns, determined by TSH and FT4 serum concentration measurement, performed on the 3-5^th^ day of life. We assessed TSH, FT4 and FT3 serum concentration on the 3-5^th^ day of life in preterm children born at our centre within three consecutive years. We assessed the incidence of hypothyroxinemia, and its cause: primary hypothyroidism, secondary hypothyroidism or low FT4 syndrome - with normal TSH concentration, its dependence - among others - on gestational age (GA), birth body weight (BBW) and being SGA. A total of 525 preterm children were examined. FT4 concentration was decreased in 14.9% of preterm newborns. The most frequent cause of hypothyroxinemia was low FT4 syndrome (79.5%). More than 92% cases of hypothyroxinemia occurred in children born before the 32^nd^ week and/or with BBW below 1500 g. Thus, every fourth child in these groups had a reduced FT4 concentration. Neonates with hypothyroxinemia were significantly lighter than those with normal FT4. In older and heavier neonates with hypothyroxinemia, serious congenital defects were observed. Neither IVH nor SGA nor twin pregnancies predispose children to hypothyroxinemia. Among newborns with untreated hypothyroxinemia in whom TSH and FT4 assessment was repeated within 2-5 weeks, a decreased FT4 concentration was confirmed in 56.1% of cases. As hypothyroxinemia affects 25% of newborns born before the 32^nd^ week of gestation and those in whom BBW is less than 1500g, it seems that in this group of children the newborn screening should be extended to measure serum TSH and FT4 concentration between the 3-5^th^ day of life. In older and heavier neonates, additional serum TSH and FT4 assessment should be limited to children with severe congenital abnormalities but not to all SGA or twins. Despite the fact that the most common form of preterm hypothyroxinemia is low FT4 syndrome, it should be emphasized that FT4 remains lowered on subsequent testing in more them 50% of cases.

## Introduction

Thyroid hormones (free thyroxine – FT4 and triiodotyronine - FT3) play an important role in the growth and maturation of many cells and tissues, not only in the foetal but also in the neonatal period. They are essential for the normal development of the brain (1, 2).

Congenital hypothyroidism is defined as a dysfunction of the hypothalamic–pituitary–thyroid axis, present at birth and resulting in insufficient thyroid hormones synthesis (3). Therefore, this condition may be caused by abnormal development or function of the hypothalamus, pituitary gland or thyroid gland and it should be detected immediately after birth, and treated with levothyroxine (LT4) in order to avoid irreversible developmental disorders (3). For many years, screening for congenital hypothyroidism has been performed around the world to diagnose primary hypothyroidism by assessing TSH in a dry blood specimen (from a heel prick), collected between the 3^rd^ and 5^th^ day of life. As secondary hypothyroidism is overlooked in this way, when financial resources are available it is recommended to measure FT4 to screen for central (i.e. secondary, due to insufficient secretion of TRH or TSH) hypothyroidism (3). While the frequency of secondary hypothyroidism is rare in full-term newborns, it is more frequent in preterm infants as the hypothalamic–pituitary–thyroid axis starts to mature by the second trimester of gestation (4–6). Therefore, the more preterm the child is, the more immature this axis is and no specific feedback regulation is observed. Thus, in these cases, both the lack of increased TSH secretion as in primary hypothyroidism and decreased TRH and TSH secretion, resulting in insufficient FT4 secretion (secondary hypothyroidism) due to the immaturity (a transient form) or disorders of the hypothalamus and pituitary gland (a permanent form), were observed (4–6).

The risk factors for hypothyroxinemia – besides prematurity – included low body birth weight (BBW), twin pregnancy, critical illness or medication during pregnancy (3, 7). Many studies discuss the validity of assessing TSH and FT4 levels in all premature babies (7, 8). In Poland, screening has been carried out for many years, involving the assessment of TSH in a drop of blood from a heel prick. So additional recommendations were published in Poland (2016) due to the need for early diagnosis and treatment of hypothyroidism in preterm newborns (9, 10). These recommendations point to the need to measure TSH and FT4 serum concentrations on the 3^rd^-5^th^ day of life (regardless of the National Newborn Screening Programme of inborn metabolism) in all preterm children, to initiate treatment with LT4 before the end of the second week of life (in case of hypothyroxinemia confirmation). According to the TSH values accompanying the reduction in FT4 concentration, primary hypothyroidism, secondary hypothyroidism or the low FT4 syndrome were diagnosed and treatment with an appropriate dose of LT4 was recommended. Therefore, we have started to implement these recommendations at our Institute, which is hospital of the 3^rd^ level of reference for taking care of newborns and provides regional and supraregional services.

Recently, the Consensus Guidelines Update was published by the European Society for Pediatric Endocrinology (ESPE) and European Society for Endocrinology (ESE). They recommend considering post-screening procedures in special categories of neonates at the risk of congenital hypothyroidism, including premature, with low birth weight, twins and sick babies (3). However, it is not clear whether all preterm and SGA neonates should be screened additionally for hypothyroxinemia, or who and when should be treated with LT4.

Thus, the purpose of the study was to determine the prevalence and causes of hypothyroxinemia in premature and SGA neonates born at the Polish Mother’s Memorial Hospital - Research Institute (PMMH-RI) in Lodz in the last three years.

## Material and methods

At the PMMH-RI in Lodz, in three (3) consecutive years: 2019, 2020 and 2021, the concentrations of TSH, FT4 and FT3 in the blood serum were assessed on the 3^rd^-5^th^ day of life, regardless of the screening test (assessment of TSH in a dry blood specimen) in children born preterm (GA <37 weeks). We collected the following data: GA, BBW, total APGAR score, single or twin pregnancy, intraventricular haemorrhage (IVH), serious congenital defects, severe illness required Intensive Care Units (ICU) and the causes of child’s death during primary hospitalisation (if it happens).

To analyze hypothyroxinemia frequency depending on GA, we divided a group of children into the following subgroups:

A: younger than 24 weeks,
B: 24_0/7_ to <28 weeks,
C: 28_0/7_ to <32 weeks,
D: 32_0/7_ to <37 weeks.

In addition, to analyze the concentration of FT4, FT3 and TSH, depending on BBW, we divided the children into the following subgroups:

ILBW - incredible low birth weight - BBW <750 g,
ELBW - extremely low birth weight - BBW ≥750 g and < 1000 g,
VLBW - very low birth weight - BBW ≥1000 g and <1500 g,
LBW - low birth weight - BBW ≥ 1500 g and < 2500 g,
NBW – normal birth weight - BBW >2500g.

The concentrations of TSH, FT4 and FT3 were measured using the electrochemiluminescent immunoassays (ECLIA) method with commercially available appropriate kits (Roche Diagnostic, Mannheim, Germany). Normal range values were as follows: for TSH: 0.7–12.0 mIU/l, with inter-assay coefficients of variation (CVs) 1.3–1.8%, for FT4: 0.89–2.2 ng/dl; and for FT3: 1.95-6.03 pg/ml with CVs 2.0–2.4%.

In accordance with Polish recommendations, we divided our group of premature babies into the following diagnostic groups (9, 10):

1. primary hypothyroidism (PHT) - if hypothyroxinemia was accompanied with elevated TSH concentration (≥12 uIU/ml);
2. secondary hypothyroidism (SHT) - if hypothyroxinemia was accompanied with reduced TSH concentration (<0.7 uIU/ml);
3. low FT4 syndrome - if hypothyroxinemia was accompanied with normal TSH concentration (≥ 0.7 uIU/ml and <12 uIU/ml).

All children with hypothyroxinemia were consulted by one of pediatric endocrinologists (all of them are co-authors of present study). According to their recommendations, in some cases, LT4 treatment was started immediately, while in others, it was recommended to repeat the test within 2-5 weeks and to take a therapeutic decision depending on the next results. L-thyroxine was recommended in doses depending on the diagnosis: in PHT – 10-15 ucg/kg, in SHT – 7-10 ucg/kg and in low FT4 syndrome: 3-7 ucg/kg (9, 10).

The data were analyzed using Statistica 11.0 software (StatSoft, Inc., Tulsa, OK, USA). The continuous variables were expressed as mean ± standard deviation for normally distributed variables. Shapiro-Wilk’s test was used to test the distribution of the variables. Correlations were evaluated using the Pearson’s test while the comparison of some count data was performed using the chi-square test. A one-way ANOVA was applied for statistical analysis with the subsequent use of a *post-hoc* test, in order to statistically assess differences between groups; Tukey’s test was selected because of the uneven amount of data in individual groups. p < 0.05 was accepted as significant value.

The diagnosis of PHT has been confirmed in all the children by a typical screening test from heel. Due to the very high concentration of TSH and very low FT4 and FT3 (the results are shown in **Table 1**), those cases were excluded from the comparative analyses concerning TSH, FT4 and FT3 results.

**Table 1:** Mean (± SD) values of TSH, FT4 and FT3 concentrations in newborns with hypothyroxinemia in individual subgroups, according to TSH values concentration.

	Primary hypothyroidism	Secondary hypothyroidism	Low FT4 syndrome	P=
No of children	3	13	62	
TSH (uIU/ml)	56.35 ± 42.69	0.47 ± 0.20	3.17 ± 2.09	0.000000
FT4 (ng/ml)	0.38 ± 0.18^*^	0.59 ± 0.15	0.62 ± 0.15^*^	0.032
FT3 (pg/ml)	0.69 ± 0.51	1.04 ± 0.40	1.23 ± 0.40	0.056

Data marked with (*) differ by p <0.01.

## Results

### The prevalence of hypothyroxinemia in preterm newborns

A total of 525 preterm newborns (246 female and 279 male) were examined. In 78 (14.9%) of them, FT4 concentration was decreased, while in 447 (85.1%) - it was within normal range. The group of children with hypothyroxinemia included 34 females (43.6%) and 44 males (56.4%). Among all preterm children, a low FT4 was confirmed in 13.8% of female and 15.8% of male children. The chi^2^ test results indicate that there were no statistical differences as regards the incidence of a decreased FT4 concentration among preterm girls and preterm boys.

### The impacts of gestational age on the occurrence of hypothyroxinemia in preterm newborns

**Figure 1:**
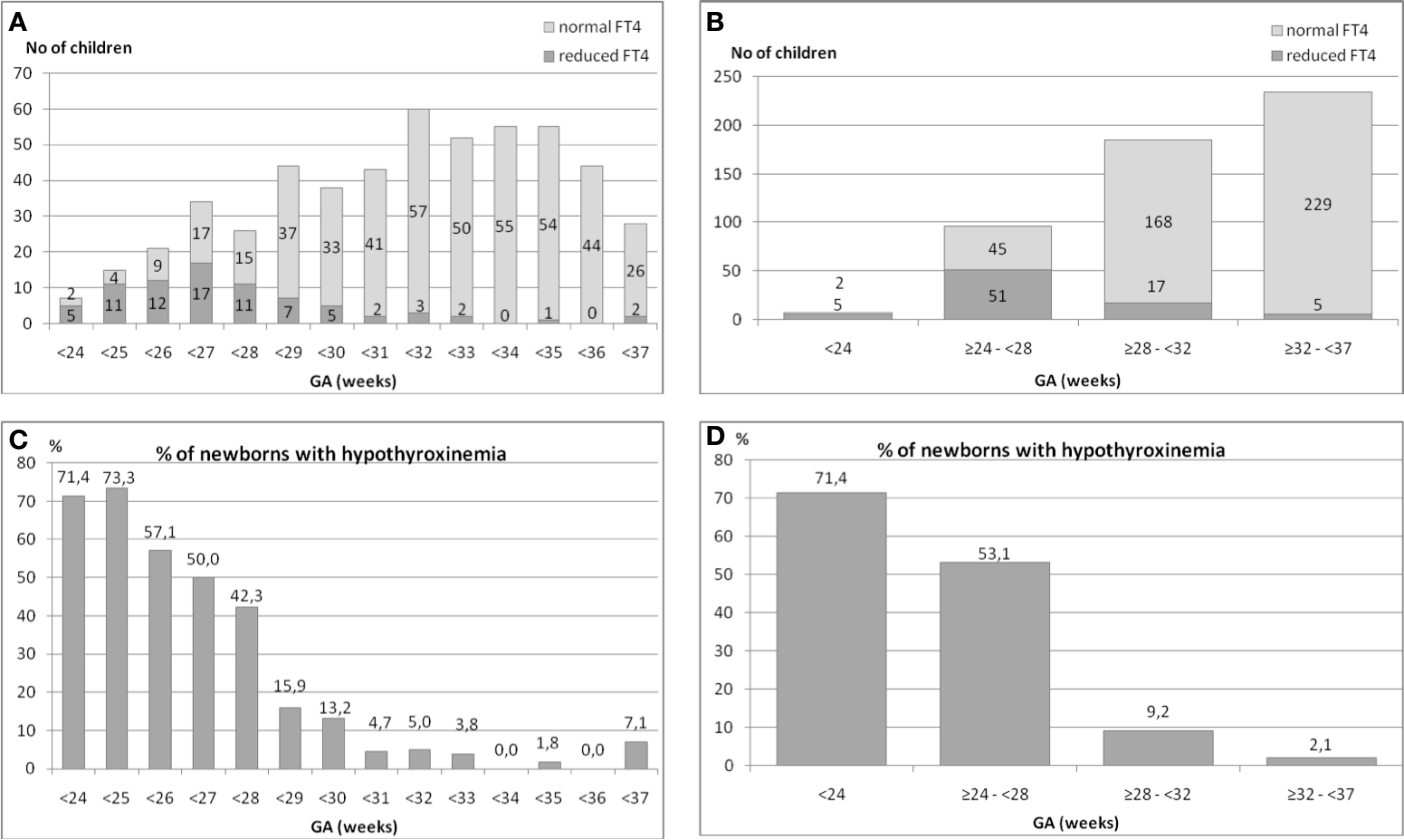
**(A–D)**. The incidence of hypothyroxinemia and normal FT4 concentration in the group of 525 preterm infants in relation to their gestational age (GA).

We analyzed the occurrence of hypothyroxinemia in the group of 525 premature infants in relation to their GA. The results are shown in (**Figures 1A**–**D**). The incidence of reduced FT4 secretion was inversely proportionate to the child’s gestational age (**Figures 1A**, **B**).

We found that in the group of children born before the 24^th^ week of GA, the incidence of hypothyroxinemia was as high as 71.4%, among those born between the 24^th^ and 28^th^ week – 53.1%, between the 28^th^ and 32^nd^ week – 9.2%, while between the 32^nd^ and 37^th^ week – only 2.1% (five cases) (**Figures 1C**, **D**).

Thus, the analysis of all preterm children born before the 32^nd^ week (n=288) showed that hypothyroxinemia occurred in 73 (25.3%) of them. That means that every fourth child in that group had hypothyroxinemia. When we analysed children born between the 32^nd^ and 37^th^ week (n=234), hypothyroxinemia was found only in 5 (2.1%) of them (chi^2^= 40.76, p<0.00005). To conclude, 93.6% cases of hypothyroxinemia were observed in children born before the 32^nd^ week of GA.

### The impact of birth body weight on the occurrence of hypothyroxinemia in preterm newborns

**Figure 2:**
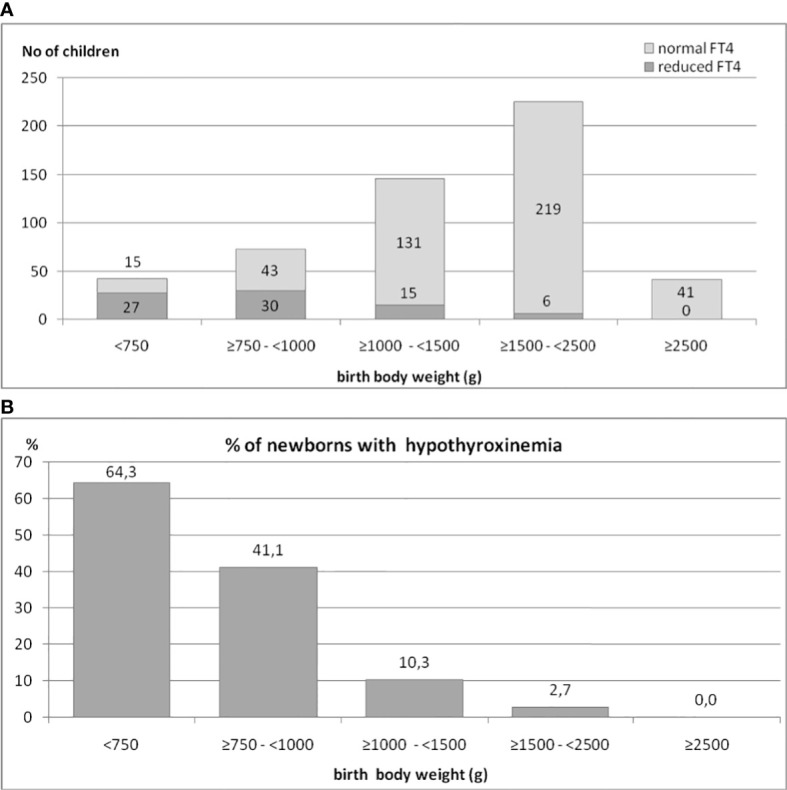
**(A, B)**. The occurrence of hypothyroxinemia in the group of 525 preterm newborns, depending on their birth body weight.

We also analyzed the occurrence of hypothyroxinemia in the group of 525 preterm newborns, depending on their BBW. The results are shown in **Figures 2A**, **B**. The incidence of decreased FT4 secretion was inversely proportionate to the child’s birth weight. We found that in the group of children born with a BBW of less than 750 g, the incidence of hypothyroxinemia was as high as 64.3%, in those with BBW between ≥750 g and <1000 g - it was 41.1%, while in those with BBW between ≥1000 g and <1500 g, it was only 10.3%. Among children with BBW between ≥1500 g and <2500 g, reduced FT4 was observed in only a few (six) cases (2.7%), while among babies weighing more than 2500g - in none. Thus, 92.3% of all cases of hypothyroxinemia were observed in children born with BBW below 1500 g (72 cases of hypothyroxinemia out of 189 children), while in children born with BBW ≥1500 g, hypothyroxinemia was observed in 6 out of 260 children (chi^2^ = 67.3, p<0.0001). In the group of babies born with BBW below 1500 g, the incidence of hypothyroxinemia was 27.6%.

### The impact of being SGA on the occurrence of hypothyroxinemia in preterm newborns

We investigated how many preterm children were born with SGA in the group of children with normal FT4 and with hypothyroxinemia. We found that in the group with hypothyroxinemia, only 5 out of 78 had been born as SGA, however, the incidence was slightly higher than in the group of preterm children with normal FT4 – 12 out of 447 (2.7%), chi^2^ = 2.94, p=0.08 (see **Table 2**).

**Table 2:** The incidence of SGA, AGA and LGA among children with hypothyroxinemia and with normal FT4 concentration (SGA, small for gestational age; AGA, appropriate for gestational age; LGA, large for gestational age; SDS, standard deviation score).

Birth weight	Hypothyroxinemia, n=78	Normal FT4, n=447	
SGA (BW SDS <-2.0)	5 (6.4%)	12 (2.7%)	Chi^2^ = 2.94; P=0.08
AGA (BW SDS ≤+2.0 and ≥-2.0)	66 (84.6%)	402 (89.9%)	Chi^2^ = 1.94; P=0.16
LGA (BW SDS >+2.0)	7 (9.0%)	33 (7.4%)	Chi^2^ = 0.24; P=0.62

We also compared the mean BBW in individual subgroups (in GA ranges), in children with hypothyroxinemia and those with normal FT4 concentration. We found that for group B: 24_0/7_ to <28 weeks, and for group C: 28_0/7_ to <32 weeks, children with hypothyroxinemia were significantly lighter (**Table 3**).

**Table 3:** Mean ( ± SD) values of body birth weight (BBW) and BBW SDS and in individual subgroups (according to GA) of children with hypothyroxinemia and those with normal FT4 concentration.

GA (weeks)	<24	≥24 and <28	≥28 and <32	≥32 and <37
No of children	5/2	51/44	17/178	5/232
BBW in children with hypothyroxinemia (g)	604.0 ± 100.39	776.0 ± 164.80	1047.64 ± 315.68	2098.0 ± 501.27
BBW in children with normal FT4 (g)	590.0 ± 56.56	931.0 ± 199.47	1358.45 ± 356.17	2030.26 ± 487.77
P=	0.864	0.000007	0.0006	0.759
BBW SDS in children with hypothyroxinemia	1.7 ± 1.82	0.47 ± 1.29	-0.32 ± 1.12	0.06 ± 1.91
BBW SDS in children with normal FT4	1.45 ± 1.03	1.25 ± 1.32	0.46 ± 1.16	0.03 ± 1.20
P=	0.864	0.004	0.008	0.952

Hypothyroxinemia was associated with a lower BBW of the child, but in many cases, the birth body weight was not low enough to meet the SGA criterion (**Table 3**).

### The impact of being a twin on the occurrence of hypothyroxinemia in preterm newborns

The analysed group of preterm children included 96 twins: 8 out of 78 children (10.3%) with hypothyroxinemia and 88 out of 447 children (19.7%) with normal FT4 concentration. Thus, the incidence of twin pregnancies was higher in the group of newborns with normal FT4 concentration than in the one with hypothyroxinemia (chi^2^ = 0.21; p>0.05).

### The impact of intraventricular haemorrhage on the occurrence of hypothyroxinemia in preterm newborns

Intraventricular haemorrhage (IVH) was found in 30 children with a decreased FT4 concentration, but in most of them, it was a low grade haemorrhage: grade I - in 4 children and grade II - in 17 children. Serious IVH occurred in 9 newborns: grade III - in 6 children and grade IV - in 3 children. In 6 of those babies, the low FT4 syndrome was diagnosed (9.7% of the entire low FT4 subgroup), and in 3 - SHT was confirmed (23.1% of the entire SHT group). So, the occurrence of serious IVH was the most frequent cause of SHT, however the chi^2^ test result for these values was not statistically significant (chi^2 =^ 1.83, p>0.05). Nevertheless, we found that the mean TSH concentration did not differ between children with IVH and without it: 2.1 ± 1.87 uIU/ml vs 2.78 ± 2.19 uIU/ml, respectively, but the mean FT4 concentration was significantly lower in children with IVH than in children without this complication (0.52 ± 0.22 ng/ml vs 0.63 ± 0.14 ng/ml, respectively, p <0.05).

### An analysis of children with hypothyroxinemia born after the 32^nd^ week and with a BBW over 1500g

The detailed data on the five children with hypothyroxinemia who were born between the 32^nd^ and 37^th^ week (and one who was born in the 30^th^ week, but with the body mass of over 1500 g), are presented in **Table 4**. In all those children, we observed some serious congenital defects.

**Table 4:** Detailed data on five children with hypothyroxinemia, born between 32^nd^ and 37^th^ week and one child born in the 30^th^ week, but with the birth weight over 1500 g (GA, gestational age; BBW, birth body weight; SDS, standard deviation score; SHT, secondary hypothyroidism; GDM, gestational diabetes mellitus).

Case	sex	GA (weeks)	BBW (g)	BBW SDS	FT4(ng/ml)	FT3(pg/ml)	TSH (mIU/l)	Type of disorder	Apgar score	death	LT4 treatment	Comorbidity
C1	f	30	1600	1.2	0.69	0.99	8.78	Low FT4 syndrome	7/8	no	no	gastrointestinal perforation
C2	f	32	1600	0.1	0.6	1.23	1.74	Low FT4 syndrome	5/6	no	no	congenital hydrocephalus, polymicrogyria
C3	f	32	2350	2.3	0.82	1.25	5.03	Low FT4 syndrome	5/7	no	no	dilated pulmonary trunk, GDM and Hashimoto’s disease - in mother
C4	m	34	2750	1.5	0.66	1.38	0.58	SHT	4/5	yes	no	cardiomyopathy, polyhydramnios, dysmorphic features
C5	m	36	1590	-2.5	0.23	0.54	0.32	SHT	7/8	no	yes	oligohydramnios, kidney failure
C6	m	36	2200	-1.0	0.71	2.14	4.37	Low FT4 syndrome	6/7	no	yes	agenesis of the corpus callosum, colpocephaly, cerebral hypoplasia

### The causes of hypothyroxinemia in preterm newborns

Depending on the TSH concentration result, among 78 children with hypothyroxinemia, 3.8% were diagnosed with PHT (n = 3), 16.7% - SHT (n = 13), and 79.5% - with the low FT4 syndrome (with normal TSH concentration) (n = 62). In all children with PTH, the diagnosis was also made on the basis of typical neonatal TSH screening (from a heel prick). The results of TSH, FT4 and FT3 levels in individual groups are presented in **Table 1**. Apart from the obvious difference in TSH concentration, which was a precondition for the division into groups, it was noticed that FT4 concentration was significantly lower in the PHT subgroup than in the low FT4 syndrome subgroup (**Table 1**).

### The causes of death among preterm newborns with hypothyroxinemia

In the analyzed group of children with reduced FT4 concentration, 8 died shortly after delivery (10.3%), 2 of them were treated with LT4, while the remaining children were not. Secondary NT was diagnosed in five (5) newborns, and low FT4 syndrome in three (3) of them. Thus, among the children with SHT, death occurred in 5 out of 13 newborns (38.5%), while among those with the low FT4 syndrome - in 3 out of 62 (4.8%). In none of those children hypothyroxinemia was the cause of death; they died due to serious concomitant illnesses.

### The usefulness of FT3 assessment in the diagnosis of hypothyroidism in preterm newborns

In addition to TSH and FT4, we also evaluated the concentration of FT3 at the same time point. Among 78 children with hypothyroxinemia, low FT3 concentration was found in 67 newborns (85.9%), normal - in 8 newborns (10.3%) and in 3 of them (3.8%) this parameter was not assessed. We found low FT3 concentrations in all children with SHT (except one), in 52 out of 62 newborns with the low FT4 syndrome (in 7 - it was normal and in 3 - it was not measured), as well as in all newborns with PHT. We did not find any differences as regards FT3 concentration among groups (**Table 1**).

We observed a strong positive correlation between FT4 and FT3 (r=0.7, p<0.05) in the whole group of the analysed children.

Among 447 newborns with normal FT4 concentration, low FT3 concentration was confirmed in 36 babies (8%). In three of them, also the TSH level was low and in those cases FT4 concentration was in the lower limit of normal range.

Therefore, it seems that FT3 measurement does not add additional information in terms of assessing the secretion of TSH and FT4 axes in newborns.

### The prevalence of hypothyroxinemia in subsequent tests performed within 2-5 weeks in preterm newborns not treated following decreased FT4 in the first test

**Figure 3:**
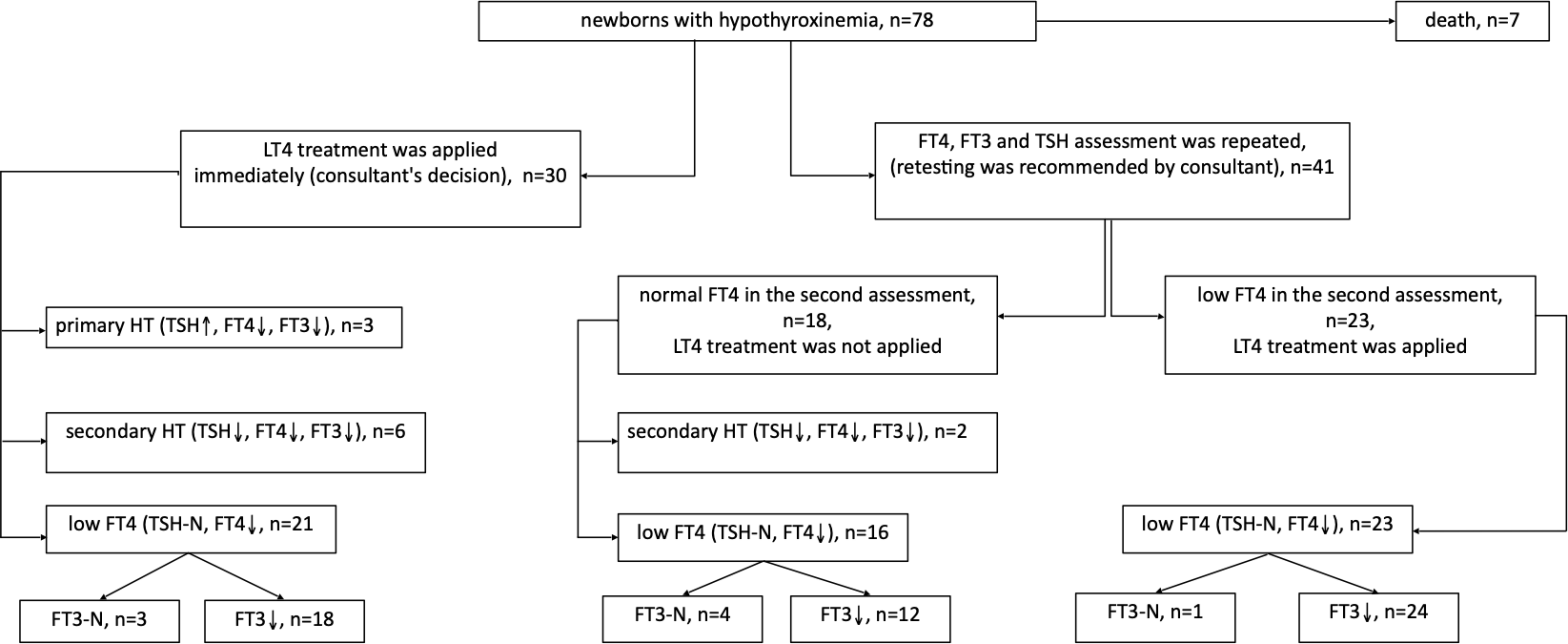
Follow up of children with hypothyroxinemia depending on the decision regarding LT4 treatment, taking into account the cause of the disease.

In 30 out of 71 children with hypothyroxinemia who were alive (seven babies died within the first 6 weeks of life, and one - included in this analysis - died in the 13th month of age), LT4 therapy was started immediately (including 3 children with PHT), while in the rest of them, the test was repeated within 2-5 weeks. In 23 out of 41 children (56.1%), the FT4 level was still decreased in the second measurement and LT4 therapy was ordered. In 18 out of 41 (43.9%) cases, normalisation of FT4 concentration was observed and children were not treated (**Figure 3**). We found no difference in the initial levels of FT4, FT3 or TSH, or GA and BBW between the groups of children with and without subsequent improvement in FT4 levels (with transient or persistent hypothyroxinemia) (**Table 5**).

**Table 5:** Mean (± SD) values of screening TSH, FT4 and FT3 serum concentrations in the group of preterm newborns with hypothyroxinemia, depending on the initial therapeutic decision and the results of tests performed thereafter (three PHT children were excluded from the analysis).

	LT4 treatment was applied immediately	FT4, FT3 and TSH assessment was repeated within 2-5 weeks		P=
		reduced FT4 in the second assessment	normal FT4 in the second assessment	
No of children	27	23	18	
TSH (uIU/ml)	2.30 ± 1.82	3.15 ± 1.97	2.84 ± 2.33	0.339
FT4 (ng/ml)	0.61 ± 0.17	0.60 ± 0.16	0.66 ± 0.14	0.505
FT3 (pg/ml)	1.19 ± 0.44	1.20 ± 0.38	1.29 ± 0.44	0.316
GA (weeks)	26.70 ± 2.82	25.83 ± 2.66	27.56 ± 2.23	0.119
BBW (g)	895.00 ± 395.96	796.09 ± 327.63	965.00 ± 274.02	0.296

## Discussion

In our study, we assessed serum TSH and FT4 levels shortly after birth, in premature infants, born over the period of three years at the PMMH-RI, in order to contribute to the discussion of whether serum TSH and FT4 assessments are worth running in addition to the routine TSH screening for all premature newborns, for the purpose of detecting hypothyroxinemia and initiating LT4 treatment as soon as possible, to avoid serious consequences related to disorders in the nervous system development (1, 2).

In 2016, based on their best knowledge, available at that time, a group of Polish experts established rules of conduct which we implemented at our centre. The main goal was to secure the proper development of the premature infants’ nervous system with the appropriate amount of LT4. Although the number of reports of transient hypothyroxinemia in premature infants and considerations regarding the principles and indications for initiating LT4 treatment is increasing, universal rules have not yet been established. Some recommendations propose that the assessment should be limited to TSH and FT4 in children born before the 32^nd^ week of gestation and with a birth body weight (BBW) lower than 1500 g, also in critically ill children or children from twin pregnancies (3, 11, 12).

We examined such newborns and found that the problem affected a large group (15%) of premature babies. However, on the basis of further analysis, it turned out that those were mainly children born before the 32^nd^ week of pregnancy and with a birth weight below 1500 g. Among those children, the incidence of hypothyroxinemia was as high as 24.6% (born before the 32^nd^ week) and 27.6% (born with BBW lower than 1500 g), comprising 92.5% of all the cases with hypothroxinemia that we detected. Therefore, we would recommend running an additional screening in that group of newborns. A similar recommendation was given by others (11–14).

In order to search for the cause of hypothyroxinemia and to establish the principles of LT4 therapy, we divided our group of children with reduced FT4 concentration into 3 subgroups, depending on the TSH result. This division was also based on our Polish recommendations, published in 2016 (9, 10). This was important because, depending on the TSH result, a different LT4 dose is recommended and, at the same time, it indicates the likelihood of the persistent or transient form of hypothyroxinemia.

However, it is debatable whether the limits of TSH are well matched and whether the distinction between secondary HT and the low FT4 syndrome is logical.

Our analysis shows that out of the three possible causes of hypothyroidism, the low FT4 syndrome is most commonly (79.5%) represented. All the cases of primary hypothyroidism were detected in a standard TSH screening and therefore did not constitute a clinical problem.

Out of 13 cases of secondary hypothyroidism, 5 children died shortly after birth, which means that their condition was very severe. Thus, it is difficult to determine whether the low TSH levels accompanying hypothyroxinemia were actually caused by secondary hypothyroidism or were due to a critical illness. This is an important issue, because the syndrome of low thyroxine and triiodothyronine in severely ill patients is not treated, and in the case of secondary hypothyroidism in premature newborns, LT4 replacement therapy is recommended. In this group, as many as 5 (out of 13) had severe IVH and those children did die - however, it did not provide a satisfactory answer to the question whether low FT4 was caused by damage to the hypothalamic-pituitary axis or a serious illness of the newborn (15, 16).

The added value of our work compared to others was the assessment of the concentration of FT3, together with TSH and FT4. We wondered if this might help to distinguish the low FT4 syndrome caused by the child’s severe condition from the immaturity of the axis. However, the concentrations of FT4 and FT3 correlated positively with each other, and there were no differences between the concentrations of FT3 in individual groups of diagnoses.

Despite the reports in the literature on a higher incidence of hypothyroxinemia in children from twin pregnancies, we did not confirm that observation in the analysed group of children (17, 18), perhaps due to small number of cases.

On the other hand, the reports on the higher incidence of hypothyroxinemia in children with SGA (9, 19) were also unconfirmed in our study. In turn, in the group of preterm newborns with hypothyroxinemia, there was a slightly higher prevalence of children born as SGA (6.4%) than in the group of preterm newborns with normal FT4 levels (2.7%). However, it was the lower birth weight that predisposed the children from the different gestational age groups to having low FT4. Thus, it was being leaner (malnourished) rather than being SGA that was a hypothyroxinemia risk factor. The data provided by Kaluarachchi et al. (20) did not confirm the higher incidence of congenital hypothyroidism in neonates with SGA after considering potential confounding factors. However, they mentioned that TSH levels were higher in newborns with SGA time compared to newborns without SGA. A similar observation was reported by Grob et al. (21) and Uchiyama et al. (22) - they emphasized that being SGA is strongly associated with delayed higher TSH, also in children with SGA born with BBW below 2000 g.

As it has been mentioned above, 92.3% of hypothyroxinemia cases are children born before the 32^nd^ week of gestation or with a birth body weight less under 1500 g. In this group, the incidence of hypothyroxinemia is about 25%. The question is which children who do not meet these criteria (older and heavier) should undergo additional TSH and FT4 serum tests in order not to overlook hypothyroxinemia. After analyzing the cases in our group, we came to the conclusion that they were children with congenital defects of various organs, both the CNS and the gastrointestinal tract, and their condition was severe. Also, Yoon et al. (23) wrote that transient hypothyroxinemia in extremely low birth weight infants is associated with mortality and composite morbidities and the initial T4 level is the most effective for predicting outcome in them. Therefore, it seems that these are children in whom additional TSH and FT4 tests are worth doing after birth.

Many reports emphasize that the hypothyroxinemia observed in premature infants is transient and that the test should be repeated (24–26). However, in our material, in 50% of the children who were not treated after obtaining the first result, hypothyroxinemia was still observed in the subsequent test, performed after a period of 2-5 weeks. This means that during the time which is crucial for the development of the brain, the concentration of FT4 was insufficient.

However, the reports of the benefits of LT4 treatment in premature infants with hypothyroxinemia are divergent (4, 14, 27, 28). In our group, it was difficult to predict in which children hypothyroxinemia would persist and in which it would be transient and FT4 would normalize without treatment. Those two subgroups did not differ in terms of gestational age, birth body weight, or the initial concentrations of FT4, FT3 and TSH. Further follow-up of children with hypothyroxinemia, both those treated with LT4 and those untreated, is being carried out, and an analysis of their condition is planned in a few years time.

Summing up, as hypothyroxinemia affects approximately 25% of newborns born before the 32^nd^ week of gestation and those whose birth weight is less than 1500 g, it seems that in this group of children, the newborn screening should be extended to measure TSH and FT4 concentrations in serum between the 3^rd^ and 5^th^ day of life. Particular attention should be paid to children with SGA and also birth weight lower than average but not meeting the SGA criterion yet), as the prevalence of hypothyroxinemia among them is the highest. It seems that in older and heavier neonates, additional serum TSH and FT4 assessment should be limited to children with severe congenital abnormalities.

Despite the fact that the most common (79.5%) form of preterm hypothyroxinemia is the low FT4 syndrome, i.e. hypothyroxinemia with a normal TSH concentration, it should be emphasized that FT4 remains lowered on subsequent testing in more than 50% of cases.

## Data availability statement

The raw data supporting the conclusions of this article will be made available by the authors, without undue reservation.

## Ethics statement

The studies involving human participants were reviewed and approved by Bioethical Committee at the Polish Mother’s Memorial Hospital-Research Institute (PMMH-RI) in Lodz. Written informed consent to participate in this study was provided by the participants’ legal guardian/next of kin.

## Author contributions

Conceptualization: MN-B, RS. Methodology: AL, MK-K, TT. Formal analysis: RS, MH, EG. Investigation: RS, MK-K, AL, MN-B, TT. Data curation: EG, MH and AL. Writing original draft preparation: MN-B, TT, RS. Writing review and editing: EG, RS and AL, supervision: AL. All authors contributed to the article and approved the submitted version.

## Funding

This research was funded by statutory funds from the Polish Mother’s Memorial Institute of Lodz, Poland.

## Conflict of interest

The authors declare that the research was conducted in the absence of any commercial or financial relationships that could be construed as a potential conflict of interest.

## Publisher’s note

All claims expressed in this article are solely those of the authors and do not necessarily represent those of their affiliated organizations, or those of the publisher, the editors and the reviewers. Any product that may be evaluated in this article, or claim that may be made by its manufacturer, is not guaranteed or endorsed by the publisher.
